# Editorial

**Published:** 1877-12

**Authors:** 


### Editorial.

The issue of the present number of the CHICAGO MEDICAL JOURNAL AND EXAMINER concludes the volume for the current year. The editorial corps, reviewing the efforts put forth to realize the aims of the *Chicago Medical Press Association* and the results actually accomplished, might with reason point, not merely to the enlargement of the present volume which has been found practicable during the year, but to the greater value of its contributions to medical literature. This last result is unquestionably due far more to the character and labors of contributors, than to the personal efforts of the editors themselves. We are content, however, to leave the consideration of these questions to our friends and subscribers, with whom, after all, the vital decision as to the merits and demerits of this JOURNAL must eventually rest.

We have to express our thanks to the various medical gentlemen who have efficiently aided us during the year. To Drs. J. E. Owens, E. W. Sawyer, D. R. Brower, J. S. Knox and W. J. Maynard, we are under obligations for the full reports from the sections of the American Medical Association in its twenty-eighth annual convention, published in our July number. To Drs. W. F. Lewis, J. S. Knox, L. W. Case, D. A. K. Steele, E. Lackner and Chr. H. Fenger, we are greatly indebted for assistance in the preparation of our monthly digest of literature. And to all who have favored us with contributions during the year, we have also to extend our acknowledgments.

It becomes our duty at this time to bid a public and cordial farewell to two gentlemen, who have been associated in the editorial management of this JOURNAL since the issue of September, 1875, when its control was first assumed by the *Chicago Medical Press Association*—Drs. J. H. Etheridge and Norman Bridge. Their efficient and valuable services have largely contributed to whatever of good has resulted from the fusion of the two separate medical periodicals formerly published in Chicago. They have done their part also in maintaining that cordial unanimity which has from the first distinguished the labors of the editorial corps, and which arouses a natural regret at this first change in its *personelle.* Their retirement is of their own choice, and necessitated by the pressure of other duties, Dr. Bridge having been entrusted with the charge of the library of the Association, and Prof. Etheridge having been appointed Secretary of the Faculty of the Medical School with which he is connected.

The editorial management will remain otherwise unchanged; and it is proper to remark here that this furnishes an illustration of one of the features of the organization which now controls the publication of this JOURNAL. The editors are elected by the Board of Directors of the Press Association, a corporate body, the constitution of which furnishes an ample guarantee as to the wisdom of their selection. While the editors, therefore, may be changed from time to time, the permanent character and value of the CHICAGO MEDICAL JOURNAL AND EXAMINER will not be thereby affected. No pains, nor expense will be spared in the future to still further increase its success and influence. It is in position to announce to the medical public, in the language, now rendered famous, of the French Marshal-President:

"*J'y suis et j'y resterai jusqu'au bout!*"

